# Grand challenges in polymer chemistry: energy, environment, health

**DOI:** 10.3389/fchem.2013.00031

**Published:** 2013-12-04

**Authors:** Pellegrino Musto

**Affiliations:** Institute of Chemistry and Technology of Polymers, National Research Council of ItalyPozzuoli, Italy

**Keywords:** polymer chemistry, nanocomposites, nanomedicine, polyelectrolytes, computational polymer science

Polymer science is a so pervasive and relevant discipline in the contemporary scenario that it is unnecessary to spend much words to emphasize its role. As a matter of fact, it has been proposed to designate our time as the polymer age, to mark its distinction from previous mankind eras dominated by a series of diverse materials (the stone, the bronze, the iron ages) and to remark that our lifestyle would be hardly conceivable without polymers. The advent and the global scale establishment of the polymer technology has shaped the world around us and has profoundly changed its perspectives, as it occurs for any revolutionary technology. Despite the astonishing achievements we have witnessed along the years, many exciting challenges remain to be faced; these are well worth to tackle because of their impact on our everyday life: examples include green polymer chemistry, environmental pollution issues, polymers for energy storage and delivery, polymers for the human health.

In the following, the research subjects considered most challenging and richer of promise will be touched upon; these will form the main focus of the Specialty Section Frontiers in Polymer Chemistry.

Traditionally, physical chemistry of polymeric systems is a field of great interest and activity, witnessed by the awarding of two important Nobel prizes (Flory, 1974; de Gennes, 1991). The extensive knowledge accumulated so far makes it a mature subject, but areas in rapid evolution still exist. Thus, although classical thermodynamic approaches are considered to be well established, major refinements are being proposed on the theme of H-bonding systems. Technologically relevant fields such as membranes for gas mixtures separation, drug delivery materials, barrier structures for food packaging, durability, and aging issues in polymers and polymer-composites, are all impacted by the ubiquitous occurrence of H-bonding interactions between the substrate and low molecular weight penetrants, especially water. Theories describing the transport properties of these systems by extending available mean-field models to include self- and cross-interactions are being actively investigated (Panayiotou and Sanchez, 1991; Panayiotou et al., 2007). The comparison of the theoretical predictions with recent experimental data on the molecular aggregates' populations, made available by spectroscopic techniques such as *time-resolved* FTIR and solid-state NMR has contributed to check and refine the theoretical approaches. Improvements are expected in the near future along the directions of classical lattice fluid theories [notably, with the introduction of Non-Random lattice fluid Hydrogen Bonding models (NRHB)] and on the side of the available schemes to treat non-equilibrium glassy polymers (Scherillo et al., 2013).

A further physical-chemistry area in turbulent evolution is computational polymer science. The fast progress in terms of computing resources, coupled with the advent of powerful, less computationally intensive methodologies (notably, the Density Functional Theory, DFT, and the associated hybrid functionals like B3LYP, which alleviated many of the deficiencies of previous formulations) provided the theoreticians (and an increasing number of experimentalists) with a tool to tackle large chemical systems, well exceeding 100 atoms. This has opened up new opportunities, especially in regard to the interpretation of spectroscopic observables (IR, Raman, UV-VIS, NMR) in terms of electronic structure methods (Castiglioni, 2007; Zerbi, 2007). This is a point of utmost interest: we are getting closer and closer to a complete understanding of the molecular spectra, which, in turn, will disclose us the wealth of molecular level information contained therein. Clearly, the well established atomistic approaches continue to dominate the scene: molecular mechanics and molecular dynamics methods, owing to their robustness and computational efficiency, still represent the work-horses to face the more demanding problems related to biopolymers, synthetic polymers both organic and inorganic, and for coarse-grained, multiscale modeling (Zang et al., 2008; Tadmor and Miller, 2011). The relevance of this research field has been recently recognized with the awarding of the 2013 Nobel Prize in Chemistry to three major players in this arena, Martin Karplus, Michael Levitt and Arieh Warshel “for the development of multiscale models for complex chemical systems” as stated by the Prize motivation. Major advancements can be foreseen in areas such as molecular mechanics and quantum mechanical calculations of intramolecular and intermolecular energies, refinement of *ab-initio* simulation of spectroscopic observables (frequency and, especially, intensity) chain-packing in the crystalline and liquid-crystalline states, rotational isomeric state theory, and in the simulation of the solid state, both crystalline and amorphous.

Synthesis and chemical modification of polymers is another area of exceptional progress. Fueled by the development of the controlled-synthesis routes [Radical Addition Fragmentation Transfer polymerization (RAFT, Barner-Kowollik, 2008); Atom Transfer Radical Polymerization (ATRP, Siegwart et al., 2012), click-chemistry (Lutz and Sumerlin, 2009) and supramolecular chemistry routes (Bertrand et al., 2012)] this field has experienced an unprecedented period of growth after years of neglect. The ability to manipulate and tune the matter at an atomic scale achieved by contemporary polymer chemists is truly remarkable: some scientists (the most enthusiastic) even suggest that, at the current state of the art, any architectural design can be achieved within very narrow limits of molecular weight and end-group fidelity. In fact, with the synthetic techniques available nowadays it is possible to develop structures (micelles) that may reversibly self-assemble according to a specific stimulus (Zhang et al., 2012), shape-memory structures (Hu et al., 2012), supramolecular assemblies mimicking the biological macromolecules and their functions (Harada and Kataoka, 2006). The current focus is no longer on structural but rather on functional polymers, with an ever increasing range of capabilities and applications. These developments are strictly related to human health issues: many of the new synthetic polymers find application as drug delivery systems (Bajpai et al., 2008; Oh et al., 2008) as molecularly imprinted polymers for clinical diagnostics (Kim et al., 2006, 2007), as intelligent targeting carriers in modern therapeutic strategies (Park et al., 2008; Wei et al., 2013), to mention only a few of the most remarkable achievements. The outcomes of these forefront researches have generated an entirely new field of activity, Nanomedicine (Park et al., 2008; Mignani et al., 2013), dealing with highly specific, molecular-scale medical intervention for treating diseases or repairing damaged tissues. Polymer-based nanomedicine, including the use of polymer–DNA complexes (polyplexes) (O'Rorke et al., 2010) polymer–drug conjugates, and polymer micelles bearing hydrophobic drugs, has emerged as one of the most attractive approaches for improving the efficacy of cancer therapeutics (Park et al., 2008). Among the most actively investigated and promising areas mention should be made of (i) stimuli-sensitive systems and their synthesis, especially in relation to polymer hydrogels; (ii) the preparation and characterization of gradient polymer surfaces for biomedical applications, that is, surfaces with a gradually varying chemical composition along one dimension; (iii) the development of increasingly efficient polymer systems for gene delivery, a field whose great promises have not been fulfilled yet, because of the many challenging issues which remain to be settled (Wong et al., 2007).

Two issues are driving worldwide research toward polymers from natural and renewable sources: the growing concern about environmental deterioration caused by the increasing production and disposal of conventional synthetic polymers, and the will to reduce the dependence of western economies from petrochemical products. Vegetable oils are one of the most readily available alternatives: they are cheap, renewable resources and the functional groups present therein can be suitably activated for polymerization (Yu et al., 2006; Raquez et al., 2010). Vegetable oils are obtained from naturally occurring plants, such as sunflower, cotton, linseed and consist, predominantly, of triglycerides. The structure of these monomers can be modified according to different strategies which, in turn led to various synthetic routes, including condensation, radical, cationic, and methathesis polymerization (Güner et al., 2006; Sharma and Kundu, 2008). Recently, the focus is being shifted toward thermosetting materials, i.e., highly cross-linked networks cured by heat, heat and pressure, and/or light irradiation. Very interesting opportunities are emerging in this area in connection with some commonly used networks such as phenolics, epoxy, polyester, and polyurethane resins (Raquez et al., 2010).

Polymers from renewable sources suffer two major drawbacks: the lesser mechanical properties and the sensible price compared to their conventional counterparts. To alleviate both these disadvantages, various blends and composites have been developed along the years: in this field improvements are required in connection to the synthesis of compatibilizers, which ensure optimum dispersion and better interfacial adhesion between the components. Furthermore, the technology of reactive extrusion, a complex and challenging area of polymer rheology, is actively investigated as a means to optimize the current products (Yu et al., 2006).

Energy conversion, storage and delivery is a further sector where polymer chemistry is playing a major role (Rabek, 1988; Page et al., 2012). Analysis of the literature shows that research efforts in this area date back from the early 1990s, but the most significant achievement have been reported in the last 10 years. It is therefore, a dynamic and up-to-date subject, strictly related to economic and social issues and capable of attracting conspicuous funding. The state-of-the-art polyelectrolytes developed so far for fuel-cell and battery applications still need important improvements, which can only be achieved through a deeper understanding of the molecular mechanisms at the background of their mesoscale behavior. The relevance of chain microstructure, chain dynamics, and nanoscale morphology on the performances of these materials cannot be overestimated. It is a highly interdisciplinary field in which the synthetic polymer chemist has to work face-to-face with the physicist and the chemical engineer, which is not always easy; this same reason, however, makes the subject so exciting and challenging. An emerging approach to overcome the current drawbacks consists in adding suitable ionic-liquids to the polyelectrolytes. These are molten salts at room temperature and possess peculiar properties such as high chemical, thermal and electrochemical stability, high conductivity and no measurable vapor pressure. Organic, semiconductor-based photovoltaic devices offer the promise of a low cost technology based on existing roll-to-roll printing techniques. However, these devices are currently limited to solar power conversion efficiencies of 3–5%. This is because of poor overlap between the absorption spectrum of the organic chromophores and the solar spectrum, non-ideal band alignment between the donor and acceptor species, and low charge-carrier mobility (Krebs, 2009). An active research area is the development of specialized polymer architectures capable of a better overlap with the sun's spectrum which can be fabricated by cheap, high throughput and high purity technologies (solution printing). Examples of such developments are ZnO nanocomposites and dendritic structures. Finally, microporous and nanoporous polymers are showing promise for energy gas storage, with the added advantage of versatility in the synthetic routes and ease of chemical modification of the resulting polymer structures (Bhoje Gowd et al., 2009; Dawson et al., 2012).

Enormous research efforts have been devoted during the last decade toward the development of nanocomposites. These were introduced in the 1990s as an extension of the classical polymer-composite concept (Pavlidou and Papaspyrides, 2008; Kango et al., 2013). The rationale behind the new approach arises from the opportunity of obtaining enhancements of properties far exceeding those achievable with macro-composites, taking advantage of the exceptional surface area of the nanofillers. However, the initial enthusiasm for the new materials was intended to be, at least partially, frustrated. After years of intense and costly research, the technology of nanocomposites has not been exploited to its full potential yet. Many opportunities still remain for improving the techniques to achieve optimum dispersion, avoiding unwanted coalescence of nanoparticles in larger aggregates. Functionalization of the filler with suitable reactants that promote dispersion and improve the interfacial adhesion is an area of intense activity (Kango et al., 2013). With respect to the preparative technologies involving the *in-situ* formation of the inorganic phase, modified sol-gel routes whose chemistry can be tailored so as to be compatible with the presence of the polymeric matrix are being actively explored (Kickelbick, 2003). Carbon-based nanocomposites represent the new research frontier on hybrid materials. Interest on these systems has flourished with the advent of well characterized and molecularly controlled carbon-based nanostructures such as fullerenes, graphene and nanotubes (Sahoo et al., 2010; Roy et al., 2012; Naffakh et al., 2013). It has been demonstrated that the incorporation of these motifs into diverse polymer matrices produces a relevant enhancement of the material's performances, most notably in terms of mechanical, thermal, electrical, and magnetic properties. Here again, the critical issues reside in the efficient dispersion of the nanofillers as well as, for the case of carbon nanotubes, their controlled alignment within the matrix. Several methods are being currently explored, among which solution-mixing, melt-mixing, electrospinning. Efficient methods for the chemical functionalization of the carbon-based nanostructures have been recognized as a key factor toward the development of high-performance materials based on this class of nanocomposites (Roy et al., 2012).

From the considerations enumerated so far emerges a discipline rich of opportunities and challenges, whose development is likely to produce significant advancements in our society. The Specialty Section Frontiers in Polymer Chemistry was born to witness, disseminate, and foster these advancements.
